# Information and education for informal dementia caregivers: the Age-it project

**DOI:** 10.3389/fpubh.2025.1683288

**Published:** 2025-11-14

**Authors:** Sara Trolese, Ilaria Chirico, Sara Santini, Georgia Casanova, Serena Cancellieri, Andrea Melendugno, Rabih Chattat

**Affiliations:** 1 Department of Psychology, University of Bologna, Bologna, Italy; 2 INRCA-IRCCS, Ancona, Italy; 3 Casa Madre Teresa di Calcutta, Opera della Provvidenza Sant'Antonio, Padova, Italy

**Keywords:** information, education, dementia, informal caregivers, E-health interventions

## Abstract

**Background:**

While there are several studies on caregivers’ needs in general, the interest towards dementia education as a mean of support and empowerment for caregivers is more recent. This article specifically aims to explore and better understand the training needs of informal caregivers of people with dementia in Italy as to inform the development of an educational tool specifically devoted to them.

**Methods:**

Informal caregivers were recruited through key informants working in the third sector and social support organizations. Two focus groups were carried out and transcripts were coded and analysed using inductive thematic analysis.

**Results:**

The sample included a total of 19 informal caregivers of people with dementia. Three themes emerged: (1) The impact of dementia on caregivers; (2) The relevance of dementia education for caregivers and society; and (3) Caregivers’ information and education needs. The general lack of information and education about dementia from the very first diagnosis affected the chance for caregivers to be effectively supported and prevent and de-escalate caregivers’ emotional distress.

**Conclusion:**

Knowledge transfer is the most proximal effect of educational interventions and should represent part of personalized multicomponent support for caregivers throughout the dementia journey.

## Introduction

Dementia is one of the major causes of disability and dependency among older adults globally, thus representing a public health priority (1). Worldwide, there are more than 55 million people with dementia, and almost 10 million new cases each year (1). Dementia is a multidomain disease that progressively impairs multiple aspects of human functioning, such as cognitive, physical, social, and emotional (2). As symptoms worsen, people with dementia show complex additional support needs, thus requiring increasing assistance. To provide it, different competences and skills are needed to effectively deal with medical issues, communication difficulties, and/or behavioral and psychological symptoms of dementia.

Recognizing the critical role of caregivers, the World Health Organization (48) in it Global Action Plan on the Public Health Response to Dementia 2017-2025 (Action Area 5) calls for enhanced support for them. Areas for action include providing accessible and evidence-based information, training programs, respite services, and other resources tailored to caregivers’ needs to improve knowledge and caregiving skills, to enable people with dementia to live in the community, and to prevent caregiver stress and burnout.

Despite it, people with dementia and their families still cannot access appropriate services and support across the world (3). In Italy, many people with dementia rely on informal caregivers (i.e., family, friends, and neighbors) to receive care. Care duties (e.g., personal care, medical care, transportation) can be multiple every day and become very physically and mentally demanding, especially when information, training, and support for caregivers are scarce (4, 5).

There is extensive literature on the multiple unmet needs of informal caregivers of people with dementia (6, 7). Evidence shows that they have more physical and mental stress than caregivers of people with a disease different from dementia (8, 9). Informal caregivers are often expected to help with the physical, practical, and emotional needs of their loved ones daily as if they were professionals. Caregivers’ needs are time-specific according to the stage of dementia. Gallagher-Thompson et al. (10) differentiated 3 stages in care partners’ needs: early (information seeking, care planning); middle (increased responsibilities, decision making); and late (anticipatory grief, adjustment to bereavement and rebuilding life after caregiving). The authors identified caregivers’ key needs for psychological support, social connection and knowledge and related health risks (psychological distress, social isolation, and disempowerment) if needs remain unsatisfied. According to a time-based nature of caregiver’s needs, it is very important that professionals prioritize caregivers’ needs at different stages while providing them with the best strategies, resources and contacts. Indeed, as the disease progresses, informal caregivers increasingly need peer support and social contact through sharing and finding solutions with others in a similar situation (11, 12). They also need some respite to take care of themselves, along with individually tailored information on dementia care (13, 14). However, their information and training needs are often overlooked and people with dementia and their families do not receive sufficient information on crucial aspects of dementia care at the time of diagnosis and afterwards (15, 16). Without prior knowledge, it is very difficult to integrate caregiving tasks into everyday life and ensure quality care.

Despite this, in recent years, especially following the COVID-19 pandemic, there has been a growing number of free resources on the Internet and social media aimed at informing and supporting people with dementia and their families (15). The use of the Internet by older adults has increased over the years, and electronic sources allow people to access health information irrespective of time and location (17). However, despite the advantages associated with easy access to information, digital training resources are often neither comprehensive nor systematized nor based on the best available evidence (18). A similar situation applies for Italy where there is a lack of personalized training programmes addressing the specific needs of people with dementia in the different stages of the disease, including communication techniques, behavioral symptom management and guidance on bureaucratic, legal and administrative issues (19). Moreover, although digital interventions are increasing, there is a lack of a multidisciplinary approach to dementia leading to missing information on crucial aspects of dementia care. Education is often provided through local initiatives relying heavily on the voluntary sector. The approach sometimes used “the one size fits all” does not allow caregivers to easily retrieve the information needed according to their actual situation and dementia stages.

Drop-out rates from online support programs represent a challenge due to the number of barriers to their access and use (20). Barriers are caregiver difficulties in finding the information needed on the Internet, or the inadequacy of information provided (i.e., lack of information or an excessive amount of it), or information difficult to understand (e.g., use of medical terms) (20). A recent integrative review (46) identified barriers at different levels: content (useless/repetitive information, lack of information specific to dementia stages, relevance of components related to culture, ethnicity or gender); format (lack of interactivity, time schedule limitations and preference for in-person education); implementation (digital divide, technical problems, fidelity assessment). In order to provide effective online education and training, it is essential to accurately evaluate the target’s needs before designing the various components while following standardized, structured-based strategies to evaluate their effectiveness.

In the field of dementia education, a standardized framework of dementia care competencies is still lacking (21). Such a framework could play a crucial role in translating emerging research findings into dementia education programs. When developing this framework, it is important to align educational efforts with the actual needs of those who receive care (22). Risks related to the digital divide, −defined as persistent inequalities in access to digital technologies, digital skills, and the ability to benefit from online resources, often shaped by socio-economic factors such as age, education, and income (23–25)- should be considered, particularly for older adults or those less familiar with technology (26).

In this context, within the project AGE-IT (PNRR PE8 “Age-It”), SPOKE 5, we aimed to design, implement, and test a prototype of an interactive AI-based information and training tool to inform and support different types of caregivers (informal, formal caregivers, and migrant care workers) involved in dementia care. This article specifically aims to explore and better understand the training needs of informal caregivers of people with dementia, both as a foundational step in the development of the tool and as a contribution to the broader knowledge on this topic. Specifically, findings from this focus group study were combined with the existing literature on (a) caregivers’ information and training needs; (b) educational interventions plus facilitators and barriers to their implementation. Once combined, the key themes for the training modules were developed following a co-design approach, and they were incorporated into our e-learning platforms for the different types of caregivers of people with dementia. Our research questions are 3: (1) What are caregivers’ training needs? i.e., what topics should be incorporated in a training program to improve the caregiving experience?; (2) What are caregivers’ access and digital skills? Access to the Internet and differences in skills and usage patterns are key determinants of online behavior; and (3) What are caregivers’ preferences about the e-learning platform layout, adoption modes and content accessibility? All this information is crucial for designing, implementing and delivering training courses with the highest chances of usability and effectiveness based on the audience’s actual needs.

## Methods

### Participants and recruitment

A convenience sample of participants was recruited between October and December 2023 through key informants working in the third sector and social support organizations active in the field of dementia. They provide information, support and psychological, social and legal assistance for people with dementia and their families while raising awareness through public outreach campaigns.

Eligibility criteria required that participants were informal caregivers of people with dementia of any type (e.g., Alzheimer’s disease, frontotemporal, mixed dementia). To be eligible, participants should have been unpaid caregivers (partners/spouses, children or other relatives) providing daily care–of any type, practical, emotional or instrumental–for people with dementia. Moreover, they could have or not have already experienced training.

Firstly, professionals and volunteers contacted potential participants via phone or met them personally to inform them about the study and ask them to take part. Once researchers get a list of potential participants, they contacted them to arrange focus group details (date, time, location). Participants did not receive any financial reimbursement for their participation. Ethical approval for the study was granted by the Ethics Committee of the University of Bologna [Ref.: 0208993]. Individuals who confirmed their role as informal caregivers of a person with dementia and met the inclusion/exclusion criteria were provided with an informed consent form. This document outlined the aims of the study and clearly stated that participation was voluntary and that individuals could withdraw at any time without any consequences.

### Procedure

A qualitative focus group design was adopted for the present study. Focus groups are particularly useful for needs assessment (27), as they facilitate self-disclosure (28) and offer a flexible approach to gathering knowledge (29). Moreover, they encourage authentic responses and allow participants to build on each other’s ideas (27).

The focus group interview guide was developed by researchers before starting data collection. Open-ended questions were formulated to prompt broader discussions of participants’ experiences, and to do it researchers drew on existing literature and their knowledge so that the topics and questions matched the research questions. Once formulated, focus group questions were reviewed by the whole research team for clarity, the order and flow of the guide and content comprehensiveness.

Two focus groups were conducted at organizations, one in Padova, northern Italy (FG1), and one in Ancona, central Italy (FG2). Each session was facilitated by two different researchers, both experienced in qualitative methods and in the field of aging, dementia and caregiving (IC: PhD, female, psychologist & SS: PhD, female, sociologist). No prior relationship was established between the researchers and participants although participants knew where the researchers worked (i.e., university and research institute) and the purpose of the study.

As mentioned previously, the focus groups aimed to identify the training needs of informal caregivers of people with dementia. This objective is part of the broader goals of the Working Package 5 of the project AGE-IT (PNRR PE8 “Age-It”), which focuses on developing an e-learning platform for both informal and formal adult caregivers, including migrant care workers, supporting people with dementia. The data collected from the two focus groups were used to identify key themes for the training modules relevant to informal caregivers and to assess accessibility features, following a co-design approach.

Each focus group lasted between 90 and 120 min. The discussions were held face-to-face, audio-recorded, transcribed verbatim, and qualitatively analyzed. For reporting in the manuscript, selected quotations were translated into English by a bilingual member of the research team and carefully checked to ensure they accurately reflected the original meaning. Participants were encouraged to share their caregiving experiences, the impact of caregiving on their daily lives, their perceived needs, particularly in terms of unmet training needs, and their preferences for engaging with an e-learning platform for dementia care. Interviewers used follow-up questions to prompt further detail or clarification when needed. The interview protocol consisted of 14 open-ended questions covering three main areas: “Training and Training Needs Area,” “Access and Digital Skills Area,” and “Platform Adoption and Content Accessibility Area.” The complete list of questions is provided in Table 1.

**Table 1:** Interview protocol.

A—Training and Training Needs Area
1. What difficulties do you face when providing care?
2. On a scale of 0 to 10, how important do you think training is for caring for a person with dementia?
3. What topics would you like to cover in a training programme? 3.1. What do you think are the most important areas to include? 3.2. What would you like to learn to improve your caregiving experience?
4. How would you prefer to learn the information you need? For example, online, in small groups, in a classroom, through videos, or via lessons?
5. Have you ever taken part in any training on how to care for someone with dementia? 5.1. If so, what topics were covered?
6. Did you find the training useful? Do you have any suggestions for improving it?
B—Access and Digital Skills Area
7. Do you find it easy to access the internet on a computer, or do you use your smartphone more often?
8. How do you mostly use your computer or smartphone? 8.1. Do you use telemedicine services for yourself or your loved ones?
9. When do you typically use these devices, and where do you usually access them from?
10. Have you ever attended an online course or used an online platform for training?
C—Platform Adoption and Content Accessibility Area
11. If you were to take an online caregiver training course, when would be the best time for you to do it, and where would you prefer to follow it from? From your home, your loved one’s home, or from work?
12. If you wanted to take an online dementia caregiver course via an e-learning platform, how would you access it? On a smartphone or tablet, or via a computer?
13. What would your ideal platform look like?
14. What would make it easier for you to use the platform?

### Data analysis

The focus group transcripts were analyzed using inductive thematic analysis (49). To ensure the reliability of the analysis, two members of the research team (IC, SS) who carried out the focus groups independently reviewed the transcripts and identified key themes and sub-themes. Text segments were coded using both deductive and inductive approaches. These codes were then systematized into a codebook and organized into sub-themes, which were subsequently grouped into overarching themes based on the internal coherence of the codes within each group. Any discrepancies between the researchers were resolved through continuous discussion and full consensus with the other study authors. Any potential researcher bias was mitigated throughout the entire study process. By engaging with the whole research team to review notes, interpretations and findings and thoroughly documenting each step of the study process, transparency was ensured, thus increasing the objectivity and confirming the accuracy of the findings. In accordance with best practices for qualitative research, we ensured that the reporting of our methodology and findings followed the COREQ-32 guidelines (50). The COREQ-32 checklist is provided as Supplementary material.

## Results

### Sample characteristics

The sample included a total of 19 informal caregivers of people with dementia. Nine participants took part in FG1, while ten participated in FG2.

Socio-demographic information was collected prior to the start of each focus group and included: gender, age, relationship to the care-receiver, level of education, arrangements, and employment status. The overall sample (*n* = 19) was composed of adult children of the person with dementia (*n* = 8), wives caring for their husbands with dementia (*n* = 6), and husbands caring for their wives with dementia (*n* = 5). The average age of participants was 65 years, ranging from 48 to 81 years. The most commonly reported level of education was completion of a university degree (*n* = 7), followed by high school (*n* = 6), middle school (*n* = 3), and primary school (*n* = 3). Most participants (*n* = 10) lived with the person they cared for. Detailed socio-demographic characteristics are presented in Table 2.

**Table 2:** Socio-demographic characteristics of the participants.

Gender	Age	Type of relationship	Level of education	Arrangements	Employment status
M	79	Husband	University	Non-cohabitant	Retired
F	63	Wife	Middle school	Cohabitant	Retired
F	63	Wife	High school	Cohabitant	Retired
M	–	Husband	Primary school	Non cohabitant	Retired
F	–	Wife	Primary school	Cohabitant	Retired
F	50	Daughter	University	Cohabitant	Employed
M	80	Husband	Middle school	Cohabitant	Retired
M	69	Husband	Middle school	Non-cohabitant	Employed
F	68	Daughter	High school	Non-cohabitant	Retired
F	74	Wife	High school	Cohabitant	Housewife
F	78	Wife	Primary school	Cohabitant	Retired
M	81	Husband	High school	Cohabitant	Retired
F	65	Daughter	University	Cohabitant	Retired
F	67	Wife	University	Cohabitant	Retired
F	56	Daughter	University	Non-cohabitant	Employed
F	57	Daughter	High school	Non-cohabitant	Retired
F	50	Daughter	University	Non-cohabitant	Employed
F	48	Daughter	University	Non-cohabitant	Employed
M	54	Son	High school	Non-cohabitant	Employed

### Qualitative findings

The inductive thematic analysis identified three overarching themes across the two focus groups: (1) The emotional impact of the disease; (2) Dementia education; (3) Training needs. A summary of the codes, sub-themes, and main themes is presented in Table 3.

**Table 3:** The analysis of the focus groups from codes to themes.

Codes	Sub-themes	Main themes
Behavioral disorders	Dementia-related disorders	The impact of dementia on caregivers
Aggression		
Sleep Disorders		
Lack of communication with the person with dementia (PWD)		
Awareness	Diagnosis	
Adjustment		
Acceptance		
Recognition		
Insidious onset		
Diagnosis		
Anger	Impact of care	
Deterioration of physical health		
Sense of danger		
Fear		
Initial self-isolation		
Loneliness		
Emotional rollercoaster		
Grief		
Caregiver burden	Need for support	
Caregiving as a job		
Available time		
Timely help		
Family care		
Services		
PwD’s routine		
Lack of knowledge	Relevance of training	The relevance of dementia education for caregivers and society
Training		
Information		
Do it yourself research		
Misinformation about the disease		
Exclusion of the caregiver		
Poor preparation of staff		
Community training		
Training for companies		
Competence and curiosity		
Family support		
Information on the web	Sources of information	
Printed information		
Unidirectional books		
Generic internet		
Contradictory information sources		
Technology as support		
Disease progression	Platform content	Caregivers’ information and training needs
Disease progression differing from person to person		
Disease progression from the early stages		
Familiarity with the disease		
Types of dementia		
Disease management by stages		
Disease management strategies		
Behavioral disorder prevention		
Personhood		
Communication of the disease		
Ageing		
Ageing and dementia		
Positivity		
Positive aspects of caregiving		
Maintenance of PwD’s autonomy		
Advice on activities for PwD		
Relational/environmental stimuli		
Socialisation of PwD		
Advice to improve the caregiver’s quality of life		
Local services		
Care management		
Home automation		
Computers	Methods and media	
Blended		
Age-adapted variety for caregivers		
Online		
Online not for everyone: digital divide		
Smartphone/WhatsApp		
Simple design platform		
Ten-point guide		
Concise slides		
Impactful videos		
Role-playing		
Practical advice		
Specific cases		

### THEME 1: the impact of dementia on caregivers

The first theme identified in the study focuses on the key challenges faced by caregivers throughout their caregiving journey, along with their emotional impact, as reported by participants. These challenges span across the management of dementia-related disorders, communication difficulties, adaptation to the diagnosis, and the need for support.

One of the most frequently cited challenges was dealing with the behavioral manifestations of dementia, particularly aggression. Caregivers described these behaviors as sudden, unpredictable, and emotionally distressing, often leaving them feeling overwhelmed and unprepared. One participant recalled a particularly distressing episode:

*“Last night, she was shouting, ‘I’ll tear you apart, I’ll break you’, Oh God, it really fills me with anguish, this is the worst situation.”*

Others similarly described the difficulty in managing such moments, especially in the absence of prior experience or guidance:

*“The most difficult thing was dealing with moments of aggression because they were sudden, because I didn’t know how to behave, so it was about managing these moments, learning to manage them.”*

Another common challenge was the difficulty of communicating with their relative with dementia. One interviewee described the emotional toll of no longer being able to engage in meaningful conversation with her husband, which led her to mourn the loss of their relationship:

*“The biggest difficulty is the lack of connection: finding yourself with a person with whom, the day before, you could talk about anything and, above all, you could get feedback. So, each of us had their own opinion about even something trivial, like a TV show, for example. And then, suddenly, there’s nothing left.”*

Another caregiver described her frustration with the communication gap, which created a constant sense of helplessness:

*“The most significant problem is the fact that she can no longer communicate, in the sense that she speaks very little and struggles to find the words. Sometimes, sorry to say, it frustrates me because we can’t understand each other.”*

Participants also reflected on the diagnosis and the emotional challenges tied to recognising and accepting the disease. One participant shared her struggle to make others acknowledge the signs of dementia:

*“I remember at the beginning, when the diagnosis was made, I realised that she was displaying some unusual behaviours, and no one wanted to acknowledge that an illness was coming. The family would say, ‘Oh, she’s just a bit distracted, she retired and doesn’t know what to do all day’, so I really needed help in this regard to say, ‘Alright everyone, let’s take a step back, because it’s not like I’m imagining these things’.”*

Acceptance was often described as a complex and ongoing process. One participant articulated the difficulty of separating the disease from the person he loves:

“I had difficulty accepting it was a disease, not *** (wife), and I was constantly measuring myself against the difficulty we call illness.”

The emotional burden of caregiving was a recurring theme, often described as a “rollercoaster” of intense and fluctuating emotions such as anger, fear, and loneliness. One caregiver summed up:

*“Besides the problem of managing the care of these people, which can sometimes be exhausting, there is also an emotional issue that those who care for them experience, and it’s no small matter. It’s truly heavy. The emotional rollercoaster that someone caring for a patient goes through is heart-wrenching.”*

The psychological toll of caregiving was considerable, with caregivers reporting a mix of fear, frustration, anger, and a persistent state of hypervigilance. Beyond episodes of aggression, other behavioral symptoms also had an emotional and physical impact. One caregiver, for example, recounted how his sleep was repeatedly disrupted:

“She was in one room, I was in another room, for example, and then she would come to me at night, waking me up even four or five times to go to the bathroom.”

Another participant described how anger would surface in response to the person’s difficult behaviors:

*“You can’t be all good with them, because the anger comes out, and that’s the hardest thing. If they were good, you would follow them with more love, but instead, you chase them with anger because of their bad behaviour.”*

Feelings of isolation were also prominently discussed. Some caregivers highlighted the lack of support from their social network, while others pointed to the emotional loneliness of hiding their true feelings to protect the person they cared for:

*“We can end up being more ill than the patient because we find ourselves alone facing a new reality. I speak for myself, as I am alone, and every day I must face new things… My husband has new problems… You are alone. Friends, close relatives, siblings don’t ask if you need anything.”*

*“It’s really hard to accept when, in front of her, I had to smile and minimise the difficulty, while inside, it was more about dealing with myself than with her.”*

Fear of being alone in the caregiving role was another source of anxiety:

*“I’m afraid of being left alone with her and not managing, because she is unable to walk and cannot move. Being alone would put me in a really tough situation. This is my fear.”*

Many participants compared caregiving to a full-time occupation, requiring constant attention, expertise, effort, and a considerable amount of time:

*“Caring for her was like a full-time job. I had to do everything for her, from getting up in the morning to preparing breakfast, washing her, and starting the day.”*

In light of these demands, caregivers stressed the importance of support systems. Day-care and residential services were frequently mentioned as crucial for emotional and practical relief:

*“The day care service and then the residential support helped. She (mother) had a group of friends here, and she felt at home, much better than at home, despite the challenges with some of the home workers.”*

Nonetheless, others expressed frustration over the lack of recognition and the difficulty of accessing services, especially in the absence of a formal diagnosis:

*“There were times when I faced difficulty getting my father’s situation acknowledged because there was no formal diagnosis. I took him for medical visits, but without a written diagnosis, they would say, ‘You should wait outside’.”*

This lack of acknowledgement was often compounded by inadequate professional preparedness:

*“During the COVID period, we couldn’t even enter, and I would think, ‘What can a person who can’t remember anything really tell you?’. Sometimes I’ve found staff to be much more knowledgeable in law than in this.”*

### THEME 2: the relevance of dementia education for caregivers and society

The second thematic area concerns the relevance of training and the sources of information accessed by caregivers throughout their journey. Participants consistently reported a widespread lack of knowledge about dementia at the time of diagnosis, which left them feeling unprepared to face the challenges ahead. Training was described not only as helpful but as essential for understanding the progression of the disease and for managing behavioral changes effectively. Several caregivers reflected on their early stages of caregiving, highlighting how inadequate preparation of health and social care staff exacerbated their feelings of uncertainty and distress.

Community-based training emerged as a crucial resource. Participants strongly advocated for broader public education on dementia, emphasizing the need to extend awareness beyond family members to professionals in various sectors, such as company staff, emergency responders, and public service workers (e.g., firefighters, police). The concept of a “dementia-friendly community” was implicitly invoked, reflecting a collective aspiration for a more inclusive and informed society.

*“In my opinion, there should be more widespread education, not just for families but also for people in offices or workplaces. It’s important that even professionals who might encounter dementia in their work are properly trained. A small mention in a safety training course could make a difference, with a link for those who want to learn more about it.”*

Many caregivers also noted that training nurtured not only practical competence but also a sense of curiosity and a proactive attitude towards learning:

*“You become competent, yes, and also more curious -you start reading online, comparing cases, asking more questions.”*

Practical exercises -such as situations and role-playing- were particularly appreciated. These methods allowed caregivers to anticipate and manage problematic behaviors (e.g., paranoia, object hiding) before they escalated. Training also helped validate caregivers’ intuitions and empowered them to modify routines or communication strategies to defuse tensions and avoid crises. Structured courses and psycho-educational groups were often described as transformative experiences, helping caregivers support not only the person with dementia but also themselves and their families:

*“That course really opened up the world to me… understanding it wasn’t just about my mother, but that there’s a trajectory and a logic to it all.”*

Nevertheless, some participants reported feeling marginalized in the clinical setting, expressing the need for more continuous and integrated learning pathways that include psychological support and opportunities for peer exchange.

The search for reliable information emerged as another central theme. Many caregivers engaged in extensive self-direct research, especially at the onset of symptoms or following a diagnosis:

*“I started looking for information myself -books, websites, anything I could find.”*

However, participants often encountered conflicting or generic information online, which led to further confusion and distress. Caregivers distinguished between generic internet sources, which they found superficial or even misleading, and more specialized information on the web, such as that provided by associations or academic platforms, which were considered more accurate and useful.

*“I read things on the internet that didn’t satisfy me because it was too generic.”*

Printed resources were also discussed, including books that narrate individual experiences of dementia. While some caregivers appreciated them for their emotional resonance, others criticised their limited applicability, noting that each case is highly individualised.

*“Books on the subject are quite one-sided. Each one seems like a biography, and most of them focus on that individual’s specific experience, rather than offering a broader understanding.”*

Some participants mentioned encountering booklets at support centers, which sometimes presented contrasting messages (e.g., *“he is no longer himself”* versus *“he is still himself”*), sparking reflection on their own relational approach to the person with dementia.

Family dynamics also played a key role in how information was processed and acted upon. Several caregivers described the emotional strain caused by relatives’ refusal to accept the diagnosis, which deepened their sense of isolation and reinforced the need for accessible, credible information early in the care process:

*“At first, nobody wanted to acknowledge there was something wrong. I needed help just to make them see I wasn’t imagining things.”*

The use of digital tools and technology to access training was positively perceived, especially by children of people with dementia who had a job. Online modules and webinars were valued by them for their flexibility:

*“My company organized lunch-hour webinars -I joined and found them really helpful.”*

These digital formats were considered particularly useful for balancing caregiving responsibilities with employment. Nonetheless, some participants expressed concerns about the lack of emotional engagement in remote formats, underscoring the importance of human connection and shared experience in the learning process.

Overall, participants stressed the importance of disseminating dementia-related information through multiple channels -online, print, and in-person- tailored to diverse learning preferences and caregiving stages. They called for an educational approach that is continuous, flexible, and grounded in a community-based model that recognizes the central role of caregivers.

### THEME 3: caregivers’ information and education needs

In discussing education needs, two main areas emerged: the content caregivers deemed essential and their preferences regarding methods and media for learning. Insights revealed a strong desire for structured, accessible, and meaningful educational resources.

Many participants expressed a need for a greater understanding of how dementia progresses, including its variability and its onset. Several caregivers emphasized the importance of distinguishing between normal aging and early signs of dementia.

*“I found it difficult to know what would happen next, what would happen tomorrow, what would happen in fifteen days. I knew that there are various stages, we all know that now based on experience… but understanding the correlation between the type of behavior, its evolution, and what to do is hard.”*

A key priority was learning about how to manage the disease across its different stages. Caregivers stressed that early access to relevant information could prevent crises and reduce stress, particularly when it comes to anticipating behavioral disturbances:

*“We realized that you can prevent certain dynamics if you recognize them early. Like with driving, you need to find a way to stop them from driving before it becomes dangerous.”*

Another recurring theme was the importance of recognizing the person beyond the disease. Training that promoted the concept of personhood, supported autonomy, and facilitated meaningful communication was viewed as essential.

Caregivers also asked for practical advice on how to engage the person with dementia in stimulating activities, such as music, storytelling, and structured routines. The value of positivity and emotional resilience was highlighted, along with the importance of learning how to access and coordinate local care services.

Regarding delivery methods, participants showed interest in both online and blended learning formats. However, children of people with dementia noted that digital tools are not universally accessible, especially for older caregivers:

*“Online is fine, but not for everyone. I’m thinking of my dad, he wouldn’t be able to manage it. He’d definitely prefer something in person.”*

Participants suggested that digital platforms should be intuitive, accessible, and age-adapted. Familiar tools such as smartphones and WhatsApp were proposed as useful channels for disseminating information and facilitating peer support.

Format and style also mattered. Caregivers appreciated short, visually engaging materials (e.g., slides, videos) and structured tools like checklists or ten-point guides. Role-playing and real-life case examples were highlighted as particularly effective, offering relatable scenarios and actionable strategies:

*“What really opened up my eyes was the course with role-playing. We acted out situations, like the woman hiding jewelry. Those scenes stayed with me.”*

*“Real examples help you recognize behaviors and make you ask the right questions.”*

Many participants reported that these approaches helped them feel more competent, confident, and emotionally equipped to care for their loved ones.

Finally, some participants suggested integrating content on technology use, such as home automation and digital health tools, to enhance safety and efficiency in care. Ultimately, caregivers advocated for practical, emotionally supportive, and adaptable training opportunities that could evolve in parallel with the progression of the disease and the needs of those providing care.

## Discussion

This study investigated the needs of family caregivers of people with dementia with attention to information and education. The thematic analysis identified three overarching themes across the two focus groups: (1) The impact of dementia on caregivers; (2) The relevance of dementia education for caregivers and society; and (3) Caregivers’ information and education needs. Each theme is described below.

The first theme concerned the diagnosis, its emotional impact on caregivers, and the need for support. It was difficult for caregivers to accept the diagnosis and to adjust to living with such a long-term condition. The most difficult issue to manage was represented by the behavioral and psychological symptoms of dementia (BPSD), causing a carousel of emotions due to the unpredictability of the disease and communication difficulties, which compromised the relationship with their relative with dementia. In such a scenario, caregivers often had to modulate their emotional reaction to dementia symptoms, thus leading to a constant state of hypervigilance and psychological suffering. Caregivers often had to deal with their own difficulties to accept the disease and their family’s ones, and they felt a profound isolation and loneliness due to everything put on their shoulders. These results corroborate the findings of a great deal of the previous work on the burden of dementia care as demanding full-time job (30, 31). Caregiving indeed implies both practical difficulties and emotional conflicts secondary to fatigue and economic problems, thus disrupting the whole family equilibrium (32, 33). In our study, some caregivers complained about the experience of feeling marginalized in the care process in favor of the patients, thus feeling their role undermined and their support needs overlooked. These findings are in line with the extensive literature on the effects of caregiving on the psychological health of those affected by the condition, thus highlighting the need to pay equal attention to people with dementia and their caregivers (34). This would be achieved by planning structured pathways of assessment and care for patients and their families based on the collaboration between various agencies, services, and health infrastructures (35). It would also have a preventive nature aimed at guaranteeing care quality, while minimizing the risk of caregivers’ burden and of its direct and indirect effects including the worsening of family relationships (34, 36).

The second theme dealt with caregivers’ need for education and valid support, due to the lack of information provided by professionals at the time of diagnosis and afterwards. Beyond diagnosis, informal caregivers received little or no information on different aspects of the disease. This supports previous studies on caregivers’ need to receive practical information on how to manage the disease, how to interact with the person with dementia and maintain a bond in the relationship and outside the family (37). Providing the much-needed personalized information through dementia stages can empower caregivers in their role while decreasing their feelings of helplessness and disorientation (10, 30). In our study, since the information provided by professionals was scarce, caregivers often used the Internet and interpreted data themselves in an uncertain way, due to the nature of data retrieved, which was often superficial or even misleading. Caregivers sometimes noticed the presence of conflicting information or unique one (i.e., not applicable to their situation), which caused increasing confusion and distress. These results are in line with the literature on caregivers’ active searching behavior on the Internet despite the presence of barriers to use such as the inadequacy of information provided (20, 37). Moreover, our findings also resonate with professionals’ lack of dementia knowledge and skills and their need to receive education to provide optimal care for people with dementia and their families (19, 38). The chance to receive appropriate information early in the care process is also important to help with the acceptance of the diagnosis, avoid caregivers’ isolation, and connect them with the available support in the territory. While there are several studies on caregivers’ needs in general, the interest towards dementia education as a means of support and empowerment for caregivers increased during the COVID-19 period (2020–2023) where the adoption of digital technologies accelerated dramatically.

Education is useful to stimulate caregivers’ curiosity towards learning about dementia instead of denying or isolating due to the idea that nothing can be done with dementia. These results support the idea that providing information empowers caregivers over the course of dementia thus promoting mastery and competence, while stimulating a virtuous learning process with tangible direct and indirect effects on all those affected by the disease (19, 30).

Education and training should be conceived as integral part of the support, rather than a local option or limited to the voluntary sector, and they should be systematized in the treatment pathways (18). As regards the Italian context, there is a lack of a systematized multidisciplinary approach to caregiver issues, and the approach “the one size fits all” does not allow caregivers to retrieve the information needed according to their actual situation (19). Our findings highlight the need for policies at community, regional, and national levels promoting the standardization of dementia care education and competencies for formal and informal caregivers. Moreover, systematic data collection and evaluation for community-based programs are missing, and, therefore, increased funding to support program development, implementation, and evaluation is necessary.

Participants in our study highlighted the importance of education for families affected by the condition as well as for the larger community and workplaces to increase knowledge, identification, and understanding of dementia and reduce the stigma based on irrational thoughts or beliefs (39). A more inclusive and informed approach towards people with dementia is therefore important to guarantee an inclusive society and avoid social inequity and exclusion of vulnerable people and their families (40). A similar approach would rely on the role of contextual factors in the adjustment to disease, while promoting the power of connection and the importance of social connectedness for the health of people with dementia and their families (41).

The third theme concerned caregiver education needs and the desired contents to be incorporated into the e-learning platform. Caregivers’ desired contents are those already identified in literature and mostly deal with the normal aging trajectories, dementia issues, stage-specific information, types of dementia, and symptoms identification (37). Informal caregivers desired to be informed on several issues, such as communication, personhood, practical care, daily activities to promote independence, specific tips on how to manage BPSD while preventing their onset or worsening, home environment, safety, and legal issues. It was very important for our participants to know about local services, when to use them, the modality of access, and entitlements. This finding is in line with the study by Soong et al. (37), while contradicts other studies which found that the caregivers’ most frequently reported need was information on the disease, followed by patient care information (6, 7). These findings reflect a shift in the focus of information needs from the disease towards available help and services, thus highlighting the need to provide service-related information since the diagnosis (37). They also reflect some peculiarities of the Italian context characterized by the lack of a well-integrated and efficient network of services working in a coordinated way, as well as the scarcity of care navigators helping patients and families to navigate through the complexity of the care system (19, 33). Other gaps in the Italian context concern the lack of professionals’ dementia-specific knowledge, as well as care staff shortages and the little time available to help patients and their families (19, 33). Regarding the lack of support, some differences occur between regions and, specifically, more services (e.g., memory clinics, day-centers, residential facilities) have been found in the North of Italy compared to the rest of the country highlighting the risk of health inequity (19).

In our participants’ view, education should also boost positivity and value positive aspects of caregiving, such as emotional resilience to counterbalance the well-known negatives of caregiving (34). This adds to the evidence on the role of self-efficacy in caregivers’ emotional and role regulation and the use of meaning-making coping as strategies to promote positive aspects of caregiving (42, 43). In our study, participants did not prefer a method of education delivery which can be in-person, online, or blended provided that it is based on users’ needs and preferences. To ensure it, it is necessary to adopt a bottom-up approach (i.e., rooted in users’ needs) and consider the risk of digital divide, which could prevent vulnerable people from using or benefiting from the use of technology (17, 44, 45). Regarding the use of technology for education and training, participants in our study valued the use of technology for its flexibility, although the lack of human contact could represent a barrier to emotional engagement (17). As previously described, caregivers’ emotional needs are several and profound and require specific attention and multi-component interventions (for example, online plus phone calls), suggesting that some caregivers can benefit from professional support and interaction with others in addition to the online support (44). Despite it, it is important to underline that research did not find significant differences between in-person and online interventions in their efficacy, which relies on the match between caregivers’ needs and preferences and the quality of intervention content and structure (15, 45).

Regarding digital interventions, few participants in our study (i.e., children of people with dementia) were unsure that their parents could easily access or use independently digital tools. These findings resonate with extensive research on the association between digital inequalities and existing disparities regarding one’s socioeconomic status, education, gender, age, ethnicity, and geographic location (24). Specifically, those traditionally disadvantaged are more likely to encounter disadvantages in their online experiences. To overcome the risk of digital divide, it is necessary to accurately evaluate caregivers’ needs and digital capabilities, thus providing the right support, face-to-face, online or mixed according to each situation (44). Moreover, this warrants the importance of stakeholders’ involvement in the design, development and assessment of online interventions (45). A co-design approach could match more caregivers’ needs and preferences while avoiding barriers to use and access of digital tools such as the inadequacy of information provided, information difficult to understand or unfriendly interfaces (15, 17, 18, 22, 44, 46). As regards the design of digital platforms, it should be intuitive and accessible for any users to avoid attrition and dropout, which occur when even a well-designed product does not match users’ needs and/or preferences (15, 22). Participants found it convenient to use smartphones and apps such as WhatsApp to retrieve information on dementia and prompt peer support. This is in line with literature which highlights the importance of incorporating the use of technology in everyday life rather than representing an additional burden on caregivers’ lives (46). Our findings corroborate the increasing evidence on the importance of using technology as mean to make life easier for caregivers, and on technology usefulness provided that the accessibility of the programme is guaranteed and the content and information match with the audience’ actual needs (15, 18, 22, 37, 44, 46).

In participants’ view, ideal contents should consist of short, visually engaging materials (slides and videos) and structured tools such as checklists or ten-point guides. Practical exercises such as role playing were particularly valued as capable of focusing on real-life situations while helping caregivers to deal with them. Moreover, group settings were perceived as useful to reduce isolation and activate common resources to deal with the diagnosis and its practical and emotional effects. A similar approach to dementia education would empower caregivers and allow them to appraise the situation as manageable, thus increasing their feelings of self-efficacy, competence and mastery as documented in other studies (15, 18). Overall, participants in our study emphasized the need for more well-structured information and training opportunities based on practical and emotional advice and flexibility through the dementia journey and the changing needs and scenarios of those affected by the condition. To be effective, education should be grounded, more practical than theoretical and continuously provided, since knowledge or care need can vary considerably depending on the stage and severity of the dementia (30, 44). A similar approach would help to guarantee the sustainability of the care sector in Italy, thus helping with the home management of people with dementia and reducing the risk of caregivers’ burn-out and institutionalization (19).

### User recommendations

Drawing on our study findings, some recommendations can be provided to create a user-friendly and effective e-learning platform for caregivers of people with dementia.

- Intuitive navigation: caregivers should be able to intuitively navigate between sections like tutorials on how to use the platform and its components, video lessons, emergency instructions, and resources without confusion. This approach can reduce cognitive load and be time effective.
- Mobile optimization: producers should design the platform to be fully responsive across devices, without losing progress or functionality. This can be important, especially smartphones and tablets, for caregivers who can only rely on mobile access.
- Personalization: the e-learning platform should allow users to personalize their learning experience. This could include options like searching content areas based on the severity and stage of dementia and skipping information perceived as less useful. Personalization can contribute to improving user experience, making the platform feel more relevant and useful to each caregiving situation.
- User-centred language: the e-learning platform should use clear, jargon-free language and maintain a supportive, empathetic tone throughout all content. Complex medical terminology should be avoided unless it’s thoroughly explained. This way, the content will be easy to understand and caregivers may feel more well supported.

Limitations and future directionsThis is the first Italian study exploring the information and education needs of informal caregivers of people with dementia within a larger project aiming to design, implement, and test a prototype of an e-learning platform to inform and support different types of caregivers involved in dementia care. Despite our findings supporting and extending previous evidence from this field, the generalizability of this study is limited by the small number of interviews and the limited representativeness of our sample. While data were collected in two regions (Veneto and Marche) which are relatively advanced regions in terms of the health and social care system in Italy, the topic of education and training in the remaining regions is yet to be explored. Future larger multi-site studies should involve purposively selected participants across different Italian regions and use gender-and age-balanced sample which could also shed light on the potential role of digital inequalities. To gain a more comprehensive understanding of the topic, professionals’ point of view may be explored. Moreover, despite the existing evidence of the positive effects of education on different domains such as caregivers’ burden, depression, and self-efficacy, more research is necessary to establish what multicomponent and/or technology-mediated intervention is most effective and in what circumstances (15, 47).

## Conclusion

Due to the increasing pressure on health and care services, information and education can effectively help guarantee the sustainability of the care sector by allowing people with dementia to receive appropriate care at home and prevent and de-escalate caregivers’ emotional distress. Knowledge transfer is the most proximal effect of educational interventions and should represent part of personalized multicomponent support for caregivers throughout the dementia journey.

## Data Availability

The raw data supporting the conclusions of this article will be made available by the authors, without undue reservation.
